# Assessing the Impact of Periodontal Therapy on Tooth Loss: A Register‐Based Longitudinal Study in Denmark

**DOI:** 10.1111/cdoe.70026

**Published:** 2025-09-26

**Authors:** Eero Raittio, Vibeke Baelum

**Affiliations:** ^1^ Department of Dentistry and Oral Health Aarhus University Aarhus Denmark; ^2^ Institute of Dentistry University of Eastern Finland Kuopio Finland

**Keywords:** cohort studies, confounding, periodontal therapy, periodontitis, tooth loss, treatment outcome

## Abstract

**Objectives:**

While regular periodontal care is advocated to reduce tooth loss considerably among periodontitis patients, evidence from observational studies is often limited to small single‐center studies. This study aims to quantify the effect of periodontal care on tooth extractions among 40‐year‐old new periodontitis patients over 20 years.

**Methods:**

A nationwide register‐based cohort study was conducted, encompassing 40‐year‐old individuals with incident periodontitis in 2001, tracked through Danish registers until the end of 2021. Receiving any periodontal care was determined annually, and the number of tooth extractions served as the annually varying outcome. G‐estimation of structural nested mean models adjusted for time‐varying confounding and loss to follow‐up was employed to estimate the average treatment effect of periodontal therapy on subsequent tooth extractions.

**Results:**

The study included 1251 40‐year‐olds with incident periodontitis in 2001. The average follow‐up from 2002 onwards was 19.1 years and amounted to 23,878 person‐years. On average, participants received periodontal care in 12.1 years (SD 6.3) and lost an average of 2.3 teeth (SD 3.5). G‐estimation showed that receiving periodontal therapy in a given year compared to not receiving any periodontal therapy reduced the number of teeth extracted in the following year by 0.04 (95% CI: 0.02; 0.06). Receiving periodontal therapy for 5 years in a row compared to not receiving any periodontal therapy was associated with an average of 0.08 (95% CI: 0.04; 0.13) fewer extracted teeth, while on average 0.62 teeth were lost in a 5‐year period (0.12 per year).

**Conclusions:**

Periodontal therapy resulted in a modest reduction in tooth extractions among 40‐year‐olds with incident periodontitis over 20 years. The effectiveness of periodontal therapy against tooth loss seems to be considerably smaller than indicated by earlier clinical studies.

## 1 Introduction

The consensus within the dental community underscores the importance of regular periodontal care in reducing adverse disease outcomes, such as tooth loss, in patients with periodontitis [1, 2, 3, 4, 5, 6]. Much of the evidence supporting this consensus comes from retrospective observational studies that, while valuable, are often limited by their scale [2, 3, 7], focusing on a single university, hospital, specialist practice, or even individual practitioners [8, 9, 10, 11]. These studies consistently report higher rates of tooth loss among patients who do not adhere to recommended periodontal treatment plans, underscoring the potential benefits of regular care [2, 3, 7].

Indeed, observational research plays a crucial role in the understanding of the effectiveness of regular periodontal care, primarily because it circumvents the ethical, practical, and financial hurdles of large and long‐term randomised controlled trials. Despite their inherent limitations, observational studies can track large populations over extended periods, providing valuable data on long‐term outcomes of long‐term treatments of relatively slowly progressing chronic diseases like periodontitis. Causal inference from observational data requires careful design and sometimes sophisticated statistical methods [12, 13, 14]. This is particularly true for long‐term and time‐varying treatments [15, 16], such as periodontal care, which are influenced by a broad set of time‐varying factors including previous treatments, socioeconomic conditions, and the severity and progression of the disease [2, 3, 7]. However, such methods have not been applied to investigate the effectiveness of regular periodontal care on reducing adverse disease outcomes, such as tooth loss [2, 3, 7].

In a previous paper [17], it has been shown, using a counterfactual longitudinal modified treatment policies framework, that reducing the frequency of receipt of periodontal therapy to no more than once every second year over a 10‐year period had minimal effect on the 10‐year risk of tooth loss in a cohort of 50‐year‐old Danes. However, these analyses do not provide estimates about average treatment effect, that is, to what extent a person receiving periodontal treatment does better in terms of tooth loss than a person not receiving it during a specific timeframe. Estimating the average treatment effect is crucial for understanding the benefits of periodontal therapy in a way that can inform clinical decision‐making and public health strategies aimed at reducing the burden of periodontitis. The average treatment effect is also a common contrast used in this research area and would thus allow easier comparisons to previous studies [2, 3, 7]. It is generally held that early and regular treatment, customised to meet the individual needs of the patient, is crucial to reduce the probability of complications of periodontitis [4, 18]. Thus, initiating treatment once periodontitis is detected at a younger age may make periodontal treatment more effective over the longer term than the earlier study suggested [17]. Therefore, the aim of the current study was to estimate the effect of receiving periodontal care on the number of subsequent extractions in 40‐year‐old new periodontitis patients over a long 20‐year follow‐up using Danish register data and robust causal inference methods.

## 2 Material and Methods

The data used for the present analyses originate from a large, register‐based prospective dynamic cohort study based on Danish register data. All permanent residents have a unique civil personal registration number, which, via the Danish Civil Registration System, allows follow‐up and linkage of individual‐level information from multiple registers. The cohort was generated by linking information from the Civil Registration System, the Educational Register, the Income Statistics Register, the National Health Insurance Service Register, and the Register for Selected Chronic Diseases. People were eligible for entry into the cohort when they reached the age of 20 years in the period from 1 January 1990 to 31 December 2021 and were permanent residents of Denmark.

The study was approved by the Danish Data Protection Agency (2015‐57‐0002) and Aarhus University (2016‐051‐000001‐914). The study was reported in accordance with the Reporting of studies Conducted using Observational Routinely‐collected health Data (RECORD) guidelines [19].

### 2.1 Sample Selection and Follow‐Up

Since 2000, private dental practitioners have had to report the number of teeth present, the number of filled teeth, and the number of decayed teeth for their patients turning 25, 40, or 65 years during the ongoing calendar year [20]. Data are reported to the National Board of Health [20] where the data have become integrated with the National Health Insurance Service Register. From the National Health Insurance Service Register, information was obtained on the dental treatments covered by the National Health Insurance scheme for subsidised dental care for all residents in Denmark between 1990 and 2021. This registry thus contains information on all patients, providers, and the services provided insofar as these are publicly subsidised and regardless of whether a person has additional private insurance or not. Because the register does not contain diagnosis codes, periodontitis was defined based on the periodontal treatment provided, as done in other studies [21, 22]. A person was considered a periodontitis case from the year onwards when the person received their first course of periodontal treatment that involved surgical periodontal treatment (1440, 1454) or subgingival/root surface instrumentation and had: (a) at least three teeth with clinical attachment loss and pathologically deepened periodontal pockets less than 5 mm (1420, 1452), or (b) one or more teeth with clinical attachment loss and pathologically deepened periodontal pocket of 5 mm or more (1425, 1430, 1453). The term “pathologically deepened periodontal pockets” was not clearly defined in the dental agreement guiding treatment code usage, but “pathologically” refers to gingival bleeding, pus, swelling, and/or colour changes [23]. In the following, the moniker “incident periodontitis” is used to describe this first occurrence of a course of these periodontal treatments. People selected this way might have been exposed to supragingival scaling and/or individual preventive services prior to becoming cases of incident periodontitis, but they had no prior periodontal treatments involving subgingival instrumentation recorded.

**FIGURE 1:**
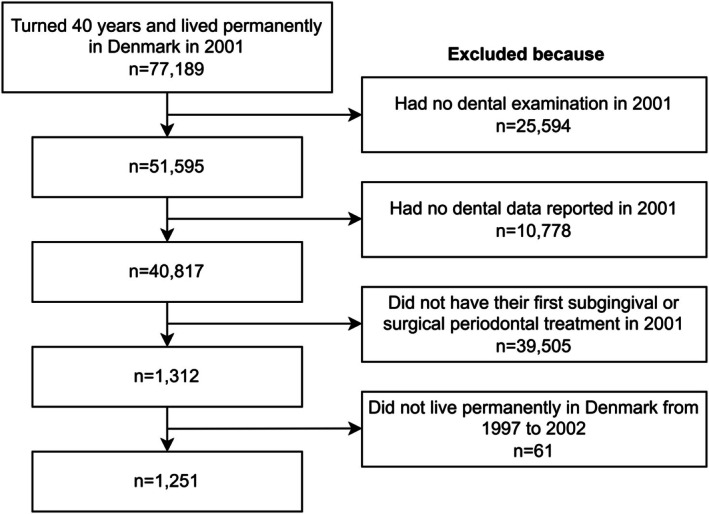
Flow chart of sample selection process.

For the purpose of this study, people were selected fulfilling all the following: (1) turned 40 years in 2001, (2) had incident periodontitis in 2001, (3) had a dental examination which included the reporting of the number of teeth present, the number of filled teeth, and the number of decayed teeth for the year 2001, and (4) had lived permanently in Denmark between 1997 and 2002. All individuals thus selected were followed from 1997 until the earliest of the following events: the end of 2021, the year of death, or permanent relocation abroad (Figure 1).

The rationale behind these criteria was to achieve a sample of 40‐year‐old individuals with incident periodontitis in 2001 who could be followed up for tooth extractions after having been observed to be periodontitis free (no recording of subgingival or surgical periodontal treatments) for a period extending at least back to 1997, and possibly back to 1990, while providing information about the numbers of teeth, filled teeth, and decayed teeth from 2001. Although no diagnostic codes were available, the findings of epidemiological studies carried out among Norwegians [24, 25] and the guidance for treatment code usage [23] indicate that all included individuals would meet the periodontitis case definition criteria of the 2018 periodontitis classification, and that most of them would have stage II‐IV periodontitis.

### 2.2 Periodontal Therapy (Exposure)

Any person selected as outlined above was defined to be exposed in the calendar year if the person had received (yes/no) any active or supportive periodontal therapy in the form of individualised prevention (e.g., individualised oral hygiene or smoking cessation advice), or any supragingival, subgingival, or surgical periodontal treatment (Supporting Information).

### 2.3 Tooth Extractions (Outcome)

The annual number of simple or surgical tooth extractions, identified via treatment codes (Supporting Information), served as an outcome. Surgical extractions involving gingival or mucosal incision, root sectioning, or bone removal were included to capture total tooth loss, thus assuming that most people had had their wisdom teeth extracted before their 40th birthday.

### 2.4 Covariates

**FIGURE 2:**
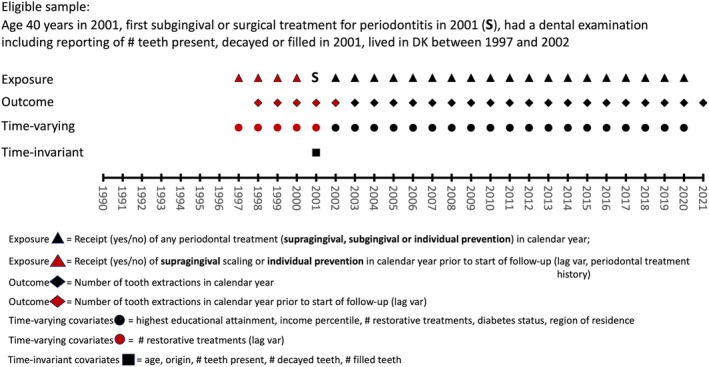
Overview of the data structure underpinning the analyses.

The time‐invariant covariates considered in the analysis (Figure 2, Supporting Information) were the 2001 recordings of gender, origin (immigrants, descendants of immigrants, Danish origin), number of teeth present (1–28), number of decayed teeth (0–28), and number of filled teeth (0–28).

Annually, time‐varying covariates (Figure 2, Supporting Information) considered were the region of residence (North Jutland, Central Jutland, Southern Denmark, Zealand, Greater Copenhagen), the highest educational attainment (eight levels), the income percentile, the number of restorative treatments in a calendar year, and Type 1 or Type 2 diabetes status, which turned from 0 to 1 in the year the patient got diabetes according to the Register for Selected Chronic Diseases (Supporting Information). Periodontal treatment history in each calendar year was captured using a variable with the following levels: (a) no periodontal treatment, (b) individualised prevention or supragingival treatment, (c) subgingival periodontal treatment of periodontal pockets less than 5 mm, (d) subgingival or surgical periodontal treatment of deeper than 5 mm periodontal pockets (Supporting Information).

### 2.5 Statistical Analysis

**FIGURE 3:**
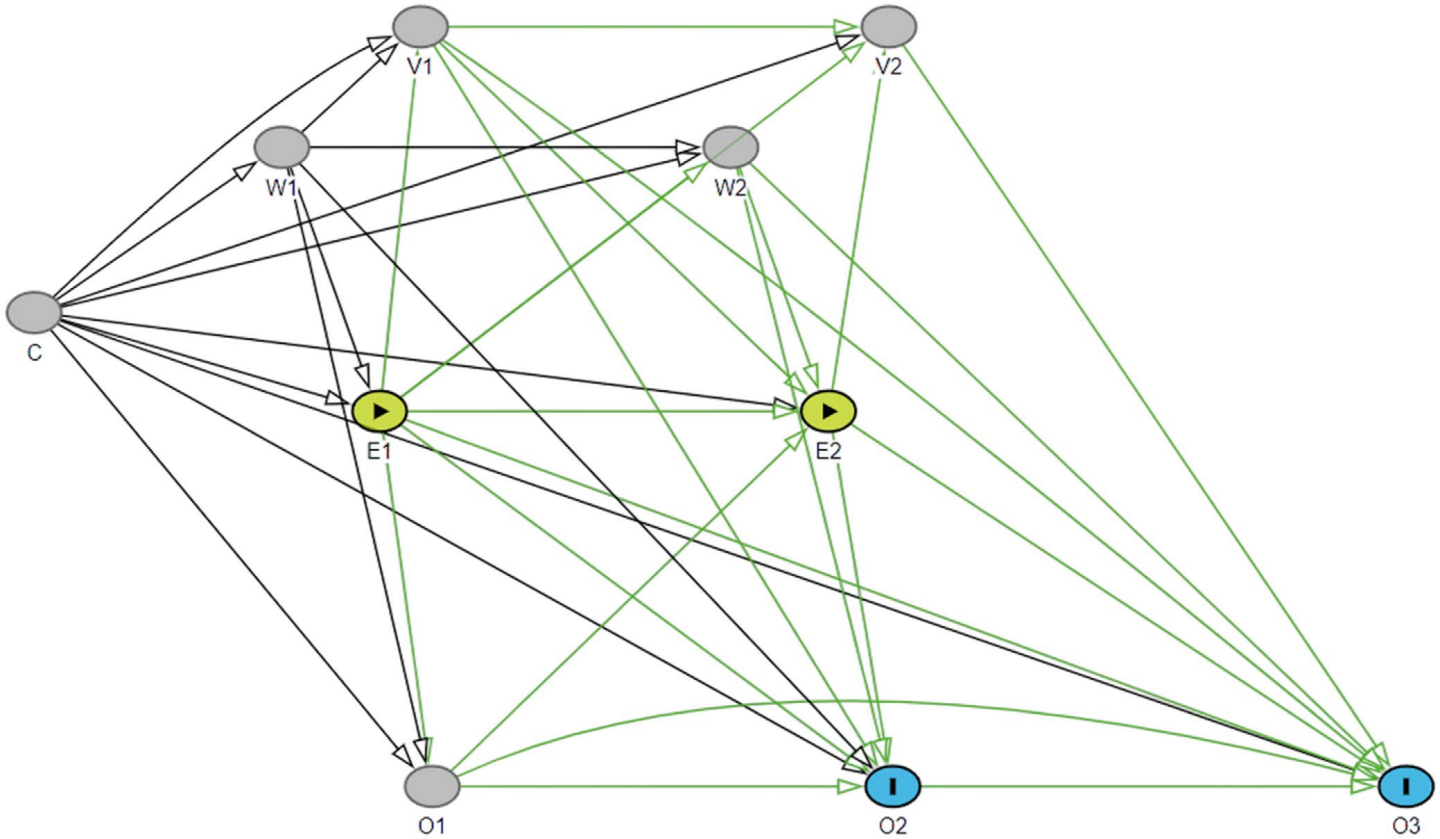
A directed acyclic graph showing a simplified version of analytical design. E is the exposure of interest; O is the outcome; C represents time‐fixed confounders; W and V represent time‐varying confounders affecting with and without lag (W) or only with lag (V). Numbers (1, 2 or 3) refer to time point. Green paths are causal paths from exposure to outcomes of interest.

G‐estimation of structural nested mean models [15, 16, 26, 27, 28, 29] was used to estimate the average treatment effect (ATE) of having received periodontal therapy in a calendar year, compared to not receiving any periodontal therapy in a calendar year, on the number of extracted teeth in the subsequent years. Traditional statistical analysis methods are not suitable for such causal inference due to the presence of time‐varying exposure and outcome, and time‐varying confounding occurring when confounding is affected by earlier exposure or outcome status, or both. This can be shown with a directed acyclic graph (DAG) (Figure 3), which illustrates a simplified version of the analytical design involving only three time points, labelled 1–3. Suppose a time‐varying binary exposure E (receiving periodontal therapy), and an outcome O (the number of tooth extractions), measured at each of the years *t* = 1, …, T. The exposure and outcome in a given year will be influenced by (1) previous exposure and outcome status, (2) time‐invariant confounders C (e.g., gender and origin), (3) time‐varying confounders W (e.g., income or education) affecting exposure and outcome with or without lag, (4) time‐varying confounders V, affecting exposure and outcome only with lag, such as treatments given at the same time as the exposure or the outcome. Note also that time‐varying confounders are affected by previous exposure and outcome status, and that from the last time point, only outcome data is used.

The effect of interest is the effect of E1 on O2, E1 on O3, and E2 on O3. The DAG presented in Figure 3 indicates that to estimate the effect from E1 to O3, one should and should not adjust for time‐varying confounders (W2, V1, V2) and previous outcomes (O2, O1) because they are both mediators and confounders on the causal pathways from E1 to O3 (green). In line with the principles of causal inference, if one adjusts for a mediator, it induces a collider bias and if one does not adjust for confounding, there will be bias from uncontrolled confounding. In such cases the use of traditional statistical methods leads to biased results, but g‐estimation may be used to handle the sitatution [15, 16, 27].

Using Figure 3 as an example, the simplified outline of the g‐estimation process [15, 16, 26, 27, 28, 29] goes as follows. Estimation proceeds by focusing in turn on each outcome time (O2 and O3) and sequentially estimating the treatment effects from previous times (E1 and E2) on that outcome. Importantly, the latest outcome, O3, can be considered as the result of the accumulation of the treatment effects from all previous time points (E1 and E2). “Peeling off” the effect of E2 on O3 from the variation in O3 allows unbiased estimation of the effect of E1 on O3 by a simple outcome (O3) regression model including E1 and all pre‐exposure variables (C, W1) while holding E2 fixed at a reference level (not treated) for everyone [15, 16, 26, 29]. To make these outcome models more robust, they are supplemented with balancing propensity scores for confounders, previous exposures, and previous outcome status for each exposure time (E1 and E2). For instance, the outcome model estimating the effect of E1 on O3 includes propensity scores to balance C and W1 by E1 status. Therefore, g‐estimation can be considered a doubly robust strategy which is not so dependent on a correctly specified single propensity score or outcome model [15, 16, 26, 27, 28, 29]. G‐estimation models can also be supplemented with weights from a censoring model to control for potentially differing drop‐out probabilities depending on previous exposure, outcome, and confounder values [15, 16, 26, 27, 28, 29].

Rather than reporting all three effect estimates from the DAG example (E1 on O2, E1 on O3, and E2 on O3)—which with 10 time points could amount to 55 effects (1 + 2 + … + 10 = 55)—one can estimate the effect of exposure on the outcome up to a specified number of time points between exposure and outcome [28, 29]. In the example, the effect estimates for E1 on O2 and E2 on O3 represent the effect of E on O one time point ahead, whereas the effect estimate E1 on O3 represents the effect of E on O two time points ahead. This offers several advantages, including improved computational efficiency by focusing on the most relevant and accurately estimated effects. It also allows for the exploration of how the effect of E on O may change over time, potentially increasing or decreasing as the interval between them widens. Furthermore, when O is a continuous outcome, the sum of these time‐varying effects can be interpreted as the average treatment effect on tooth extractions of receiving periodontal therapy at all time points versus not receiving it at any time point during follow‐up [26, 28, 29].

G‐estimation of structural nested mean models was used by applying variable specifications as outlined in the following. In line with the simplified DAG (Figure 3), the exposure was the time‐varying binary recording of receiving periodontal therapy (yes/no) in a calendar year from 2002 (E1) to 2020 (E19), and the outcome was the number of extractions in a calendar year from 2003 (O2) to 2021 (O20). Gender, origin, baseline number of teeth, baseline number of decayed teeth, and baseline number of fillings were the time‐invariant confounders (C) measured in 2001 (*T* = 0). Educational level, income, municipality/region, and diabetes status were considered time‐varying confounders with a 1‐year lag on outcome and no lag on exposure (W). The number of restorations was considered a time‐varying confounder affecting only with a 1‐to‐5‐year lag on outcome and exposure (V). Additionally, previous outcome and periodontal treatment history were allowed to affect subsequent outcome and exposure status with a 1‐to‐5‐year lag starting from the beginning of follow‐up (2002, *T* = 1) (Supporting Information). In the analyses, continuous covariates with sufficient variability—income and number of fillings—were allowed to have non‐linear relationships with the outcome and the exposure using natural cubic splines. Additionally, censoring weights for death or move abroad before the end of follow‐up were generated using the same adjustment sets.

Utilising the above‐mentioned model specifications and the gesttools [28] package in R (Supporting Information), g‐estimation of structural nested mean models was conducted to estimate the time‐varying average treatment effect (ATE) of receiving periodontal therapy at any exposure time on the number of extracted teeth in the ensuing one to 5 year period. This represents a causal contrast between a counterfactual situation where everyone in the study population would have received periodontal therapy in a calendar year compared to a counterfactual situation where no one would have received any periodontal therapy in a calendar year. Three different outcome specifications were used. First, the number of extractions within a calendar year was treated as a continuous variable and employed linear regression in the g‐estimation. Even though the linear modelling of the number of extracted teeth in a calendar year most likely violates the assumptions underpinning linear regression, it allows the estimation of what would be the average treatment effect (ATE) of receiving periodontal therapy each year over a 5‐year period compared to not receiving periodontal therapy in any year over the 5‐year‐period [28, 29]. This represents a causal contrast between a counterfactual situation where everyone in the study population would have received periodontal therapy for 5 consecutive years compared to a counterfactual situation where no one would have received any periodontal therapy for the 5‐year period. Analyses were also run considering the outcome as a count variable, or as a dichotomized variable, i.e., having at least one tooth extraction within a calendar year. In both cases, gamma regression with a log link function was used [26, 28]. In these cases, the effect modelled is either the ratio of the expected numbers of tooth extractions within a calendar year in one to 5 years ahead or the ratio of the probability of having at least one tooth extraction within a calendar year.

95% confidence intervals (CIs) for the estimates were generated by bootstrapping the dataset 500 times. In addition to basic descriptive statistics, the findings from the propensity score model are reported, indicating how the included variables were associated with receiving periodontal therapy in a calendar year.

### 2.6 Sensitivity Analyses

**FIGURE 4:**
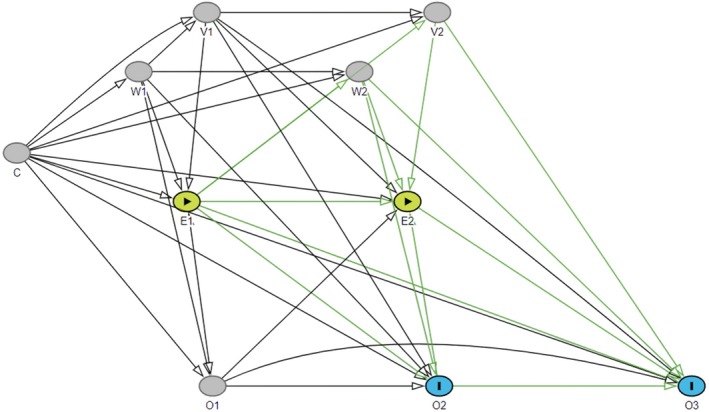
An alternative DAG. E is the exposure of interest; O is the outcome; C represents time‐fixed confounders; W and V represent time‐varying confounders affecting with and without lag (W) or only with lag (V). Numbers (1, 2 or 3) refer to time point. Green paths are causal paths from exposure to outcomes of interest.

As the actual temporal relationships between the dental treatments received within a given year were not determined, the DAG in Figure 3 represents just one possible data generation process. A sensitivity analysis was therefore conducted based on an alternative DAG, in which the arrows from E to O and from E to V were reversed in each year (Figure 4). This means that instead of assuming that other treatments (V) and extractions (O) in the same year are caused by periodontal care (E), they are considered determinants of receiving periodontal care in the same year. As an example, this could occur when a patient visits a dentist due to symptoms or other acute needs, which are treated (e.g., with extractions or fillings), and then periodontal treatment needs are detected and taken care of. Therefore, they should be included in the models estimating the effects originating from the same year and not omitted as done in the primary analyses. These analyses served to indicate the sensitivity of the results to the specification of the unknown temporal relationships among recordings from the same year.

OpenAI GPT‐4.1 was used for language refinement during the manuscript preparation.

## 3 Results

The final sample of 40‐year‐old individuals with incident periodontitis in 2001 included 1251 persons (Figure 1), who were followed for 30 133 person‐years from 1997 to 2021, or up to censoring due to death or moving abroad after 2002. The average duration of the follow‐up from 2002 onwards was 19.1 years and amounted to 23 878 person‐years.

The vast majority were of Danish origin and had a complete dentition (28 teeth) at baseline (Table 1). At baseline (2001) the median number of filled and decayed teeth was 12 (IQR = 8–16) and 0 (IQR = 0–2), respectively. The sample had a higher average income than the general population (median income percentile over 50), and over two‐thirds of the sample had vocational education as their highest educational level.

**TABLE 1:** Characteristics of the analysed sample in 2002 and 2020.

Baseline characteristics	2002	2020
	*n* = 1251	*n* = 1140
Men, *n* (%)	686 (55)	610 (54)
No. of teeth, median (Q1, Q3)	28 (28, 28)	28 (28, 28)
No. of filled teeth, median (Q1, Q3)	12 (8, 16)	12 (8, 16)
No. of decayed teeth, median (Q1, Q3)	0 (0, 2)	0 (0, 2)
Danish origin, *n* (%)	1149 (92)	1047 (92)
**Time‐varying characteristics**		
No. of restorations in a calendar year, median (Q1, Q3)	0 (0, 1)	0 (0, 1)
Income percentile, median (Q1, Q3)	69 (50, 84)	68 (43, 82)
Type 1 or 2 diabetes, *n* (%)	13 (1.0)	104 (9.1)
Educational level, *n* (%)		
Primary school	352 (28)	263 (23)
Vocational	508 (41)	481 (42)
Short‐cycle higher education	70 (5.6)	69 (6.1)
Medium‐cycle higher education	152 (12)	165 (14)
Other (4 levels)	169 (13)	162 (14)
Region, *n* (%)		
Northern Jutland	106 (8.5)	108 (9.5)
Central Jutland	288 (23)	261 (23)
Southern Denmark	256 (20)	242 (21)
Capital region	408 (33)	342 (30)
Zealand	193 (15)	187 (16)

*Note:* Q1, Q3 = 1st and 3rd quartile.

**FIGURE 5:**
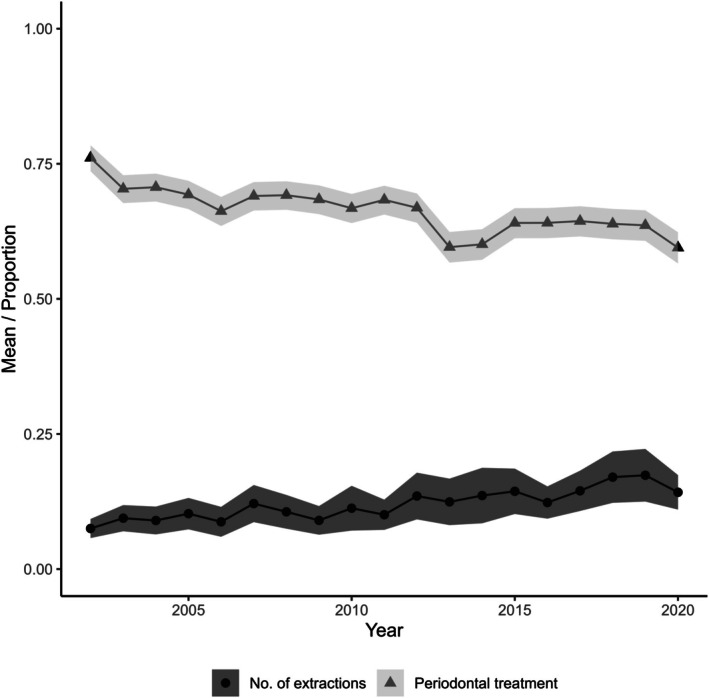
The average number of extractions within a calendar year from 2003 to 2021 (outcome) and the proportions of individuals who received periodontal treatment within a calendar year from 2002 to 2020 (exposure).

Figure 5 shows that 60%–75% of the individuals received any periodontal care in the calendar year from 2002 to 2021. The percentage slightly decreased over the years. Supragingival treatment was the most common type of periodontal care provided to individuals throughout the study period, although its prevalence decreased from over 50% to under 40%. Around 25% of individuals received subgingival treatments of periodontal pockets less than 5 mm in a calendar year during the follow‐up (Table S1). The percentage of those who received subgingival treatments for periodontal pockets 5 mm or more during a calendar year increased from around 15% to 25% during the study period. On average, a person had 12.1 (SD 6.3) years in which they received periodontal care.

The average number of extractions in a calendar year increased from approximately 0.09 to 0.17 during the follow‐up (Figure 5), being 0.12 on average. Over follow‐up, 2814 teeth were lost, amounting to an average of 2.3 (SD 3.5) teeth lost per person.

Receiving periodontal therapy within a calendar year was strongly associated with periodontal treatment history in the previous 5 years, e.g., having received subgingival or surgical periodontal treatment of periodontal pockets of 5 mm or more in the previous year was associated with 8.11 higher odds of receiving periodontal therapy next year (propensity score model, Table S2) compared to those who did not receive any periodontal therapy in the previous year. Having received extractions in the previous 5 years was associated with a slightly lower probability of receiving periodontal therapy in the calendar year. Other variables associated with receiving periodontal therapy within a calendar year were income, region, education, gender, origin, and the baseline number of decayed teeth.

**FIGURE 6:**
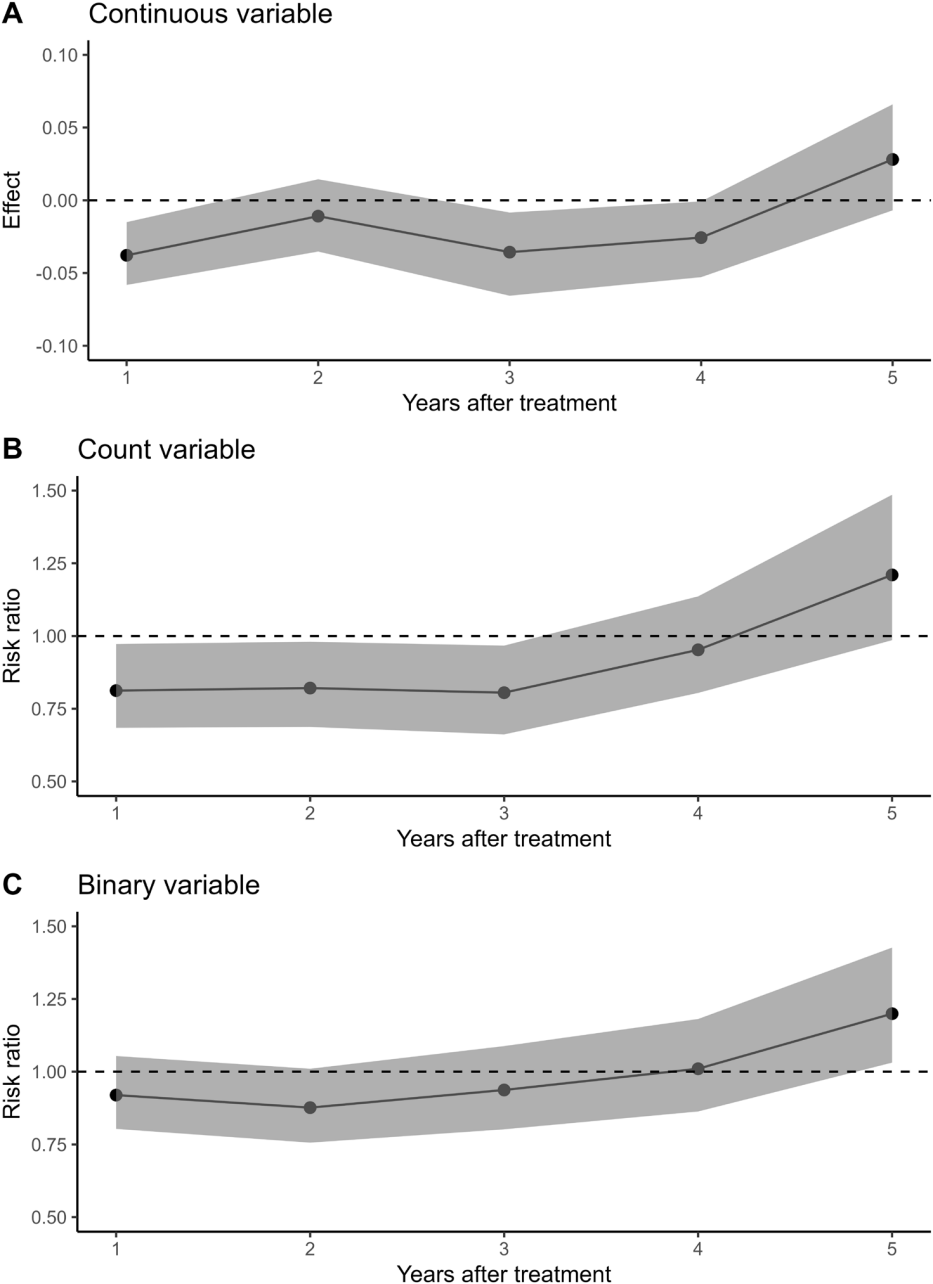
The effect (non‐treated versus treated) of receiving periodontal treatment within a calendar year on the number of extracted teeth (A, B) or the probability of at least one tooth extraction (C) one to 5 years later.

G‐estimation showed that receiving periodontal therapy in a calendar year resulted in the number of extracted teeth in the following year being lower (effect = −0.04 teeth, 95% CI: −0.06; −0.02, Figure 6A) than seen among those not receiving periodontal therapy. The effect on the number of extracted teeth 2 years later was slightly smaller than the effect on the number of extracted teeth 3–4 years after treatment, and the effect of receiving periodontal therapy in a calendar year on the number of teeth extracted 5 years later was close to null (effect = 0.03, 95% CI: −0.01; 0.07, Figure 6A). Because these effect estimates originated from the linear models, it can be estimated that receiving periodontal therapy in 5 years in a row compared to not receiving any periodontal therapy is associated with an average of 0.08 (95% CI: 0.04; 0.13) fewer extracted teeth.

On the risk ratio scale, the effect of periodontal therapy amounted to a risk ratio of 0.92 (95% CI: 0.80; 1.05), indicating a lower risk of receiving a tooth extraction (Figure 6B) and a 0.82 (95% CI: 0.68; 0.97) lower ratio of the expected number of tooth extractions in the year following periodontal therapy (Figure 6C) among the treated. The small protective effect of receiving periodontal therapy within a calendar year lasted 3 years (Figure 6B,C), and the effect was close to null or even associated with a higher risk of tooth extractions 5 years later.

Results were practically identical when variables were operationalised in line with the alternative DAG (Figure S1).

## 4 Discussion

The findings of this register‐based study indicate that the effect of receiving periodontal therapy in a calendar year on average reduces the number of extracted teeth by 0.04 (95% CI: 0.02; 0.06) teeth in the following year among 40‐year‐olds with incident periodontitis who have been followed for an average of 19.1 years. This reduction corresponds to a ratio of the expected number of tooth extractions in the year following periodontal therapy of 0.82 (95% CI: 0.68; 0.97), indicating that if the untreated would expect one tooth extraction, the corresponding figure would be 0.82 for the treated. Similarly, the risk ratio for tooth extraction in the year following periodontal therapy was 0.92 (95% CI: 0.80; 1.05), indicating an 8% lower risk among the treated than among the untreated. The protective effect of periodontal treatment seemed to disappear after 3 years, and it can be estimated that receiving periodontal therapy for 5 years in a row compared to not receiving any periodontal therapy was associated with an average of 0.08 (95% CI: 0.04; 0.13) fewer extracted teeth. Even though the current study focused more clearly on periodontitis patients by including only people with incident periodontitis, and utilized twice longer follow‐up and different causal inference methods, these findings align well with previous findings [17] that restricting the frequency of periodontal therapy to no more than once every second year among a random sample of 50‐year‐olds did not have a practically significant impact on the cumulative incidence of tooth extraction.

Despite robust causal inference methods and long‐term, rich, and nationally representative Danish data being used, this study is not without limitations. Considerations on the availability of the dental status data led to a focus on the 2001 cohort of 40‐year‐olds. While 40‐year‐olds constitute a slightly younger age group than seen in most of the smaller retrospective cohort studies, where the mean age at diagnosis/treatment initiation ranges from 41.2 to 53.7 [3] years or from 39.1 to 55.7 [11], it is not obvious that this should have an impact on the disease severity at enrollment. One would rather expect that the slightly younger‐than‐commonly‐seen patients would affect the size of the study population. A caveat might be that periodontitis patients studied in university centers or specialist practices could represent patients referred from general practice owing to their particularly complex case presentations and thus represent a relatively more severely affected subgroup than is typical in the general population. Most often, it is not clear from the publications how participants were recruited, and this may clearly hamper the interpretation and generalizability of the results. The advantage of the present study is thus its status as a truly population‐based study representing all patients who have been in contact with the dental care system for adults.

Another limitation originates in the fact that the present analysis concerns users of the dental health care system for adults. It is well known that increasingly larger proportions of the adult Danes do not participate in regular dental visits, and the dental visiting frequency is decreasing among the younger age groups (below 65 years) [30]. It is also known that the people least likely to engage in regular dental visits have lower education and a more strained personal economy than the users of dental health care [30]. It is therefore no surprise to us that the income in the sample analysed was higher than the income in the general population, as evidenced by the sample median income percentile.

A third limitation may be seen to lie in the definition used of the exposure variable periodontal therapy, as any active or supportive periodontal therapy in the form of individualised prevention or any supragingival, subgingival, or surgical periodontal treatment. Hence, each of these components is likely to have vastly different effectiveness when applied to the same periodontal condition. However, the basic tenet of the present analysis is that the dental practitioner possesses competences that allow them to customise the different treatment components to meet the needs and demands of their patients. The scope of the present analysis was not an investigation into the relative effectiveness of different forms of periodontal therapy on tooth loss, but rather an assessment of the effect of the entire periodontal therapy package in the hands of general dental practitioners. Moreover, because the methods used here rely on estimating average treatment effects using causal contrasts between “treat everyone” and “treat no one” strategies, the exposure definition used is one of the most applicable. Hence, treating people with only a need for individualised oral hygiene advice using periodontal surgery could be considered unethical and thus not a very meaningful causal contrast (treat everyone surgically vs. treat no one surgically).

Concerns could also be raised against our use of any tooth extraction as the outcome of interest, as it can be argued that not all tooth extractions have been caused by periodontitis. However, assigning a valid cause of a tooth extraction is extremely difficult because a tooth extraction necessarily involves a decision by a dentist and is influenced by a number of non‐disease factors, such as the overall treatment plan, treatment difficulty, and the financial situation of the patient [31]. The use of a disease‐specific tooth loss outcome is therefore inherently prone to numerous biases, and in line with the recommendations in general medicine [32], all‐cause tooth loss is much preferred for valid analysis results.

Because treatment and dental health records in the National Health Insurance Service Register are mainly gathered for administrative purposes, there may be variation in how the number of teeth, number of filled teeth, and number of decayed teeth are recorded, or in how the legal guidance and definitions of the indications for the treatment codes have been applied. It is therefore not possible, based on the present analysis, to infer what would have been the outcome if the quality, contents, and structure of diagnosis and treatments had been equivalent to that provided by university or specialist care professionals who do not represent the average care given to the average periodontitis patient. However, because the potential misclassification or measurement error is not likely to depend strongly on the exposure or outcome, the implications are presumably relatively small for the results of the analyses. Even though sophisticated causal inference methods were used, all observational research, particularly register‐related research, is always prone to some degree of unmeasured confounding. Unmeasured factors related to tooth loss and adherence to periodontal therapy may have biased some of the effect estimates towards null and some away from null. For instance, smoking, which is the strongest cause of periodontitis, seems to be associated with higher tooth loss and with not receiving treatment [2], and this may have biased the estimate towards protective effects of periodontal therapy. Had it therefore been possible to adjust for smoking status or history, the effects of periodontal therapy on tooth loss would probably have been smaller (closer to null). Additionally, the adjustments made in the analyses may not fully account for the varying disease severity, even though 5‐year periodontal treatment history was adjusted for. This could influence both the likelihood of receiving periodontal treatment and experiencing tooth loss, potentially biasing the estimates towards no effect [2]. It might have been desirable to be able to use actual measures of disease severity in the analyses, e.g., using the most recent classification system for periodontitis [33] or information from periodontal chartings. On a slightly different note, a methodological challenge may have arisen from the fact that having a tooth extraction was a relatively infrequent event in the study population, and this results in an outcome variable with a rather skewed distribution, which is suboptimal for common statistical models.

When interpreting the findings, it is important to bear in mind that periodontal therapy was defined as receiving any periodontal therapy within a calendar year. Clearly, this reflects the visiting intervals deemed appropriate for periodontitis patients by current dental community standards [4, 5]. However, this exposure definition may entail treatment patterns that some could consider suboptimal, e.g., periodontal therapy delivered at almost 2‐year intervals (visit in January, and then next in December in the following year). Furthermore, there is no guarantee that the treatments provided align with contemporary standards [4, 5]. Accordingly, the treatment effect estimates may be biased towards null to an unknown extent. However, the implications of this underestimation are likely to be quite small because only a minority of people seem able or willing to receive periodontal therapy in the long term as recommended. Hence, Echeverria et al. [2] concluded that “During supportive periodontal treatment, most patients abandon the recommended regimen during the first or second year”, and Knight and Thomson [29] found that “In the real world, even high‐fee‐paying specialist practice patients often do not follow professional advice; the reported long‐term retention of patients on maintenance programs in private periodontal practice is low (40% in the first year with an attrition rate of 10% every year thereafter)”.

Notwithstanding these limitations, the reported treatment effect estimates provide important insights about the real‐world effectiveness of periodontal therapy on tooth loss in Denmark. Because there are no quality and long‐term randomised clinical trials on the topic [1], analysing rich and long‐term routinely collected data has the potential to provide the meaningful evidence needed for clinical and regulatory decision‐making. It is also necessary to evaluate and confirm the findings from smaller‐scale clinical investigations, frequently conducted in hospital or specialist settings [3], which may not be well generalizable to the populations and settings where most of the periodontal care is delivered.

Apart from a recent study [17], the few earlier studies [34, 35, 36] investigating the effectiveness of periodontal care on tooth loss using routinely collected data have had considerable limitations regarding the data, study design, and analysis techniques required for credible causal inference using routinely collected healthcare data [14, 37, 38]. Using health insurance data, a Taiwanese study [35] compared the 1.5‐year cumulative incidence of tooth extractions between groups receiving either conventional or more comprehensive periodontal treatment courses. After adjusting for baseline demographic, socioeconomic, selected health conditions, and clinic attributes, those who received a comprehensive periodontal treatment course were less likely (OR: 0.83; 95% CI: 0.81; 0.85) to have a tooth extraction over a 1.5‐year follow‐up. Using health insurance data, a German group [34] investigated the 4‐year cumulative incidence of tooth extractions among a group that had received periodontal care during the previous 4 years and a group that had not received such care. The groups were matched based on age, sex, and region. Those who had received treatment had slightly higher 4‐year cumulative tooth extraction incidence (36.2% vs. 27.5%). Another German study [36] compared tooth loss rates across three cohorts: periodontally treated patients from routine health insurance data, a population‐based cohort (including both periodontally treated and untreated), and periodontally treated patients in a university hospital setting. The study examined the unadjusted average annual tooth loss rates and the incidence rates of at least one tooth extraction across these cohorts. Results showed that the population‐based cohort, regardless of whether receiving periodontal care or not, had lower tooth loss rates compared to both the register‐based (insurance data) and university hospital cohorts. Comparing these conflicting findings to the present results is difficult, if not impossible, but it is worth mentioning that this and an earlier study [17] represent a significant methodological improvement in the analysis of routinely collected healthcare data in periodontology.

What then emerges from observational studies on the effectiveness of receiving periodontal care on tooth loss in more clinical settings? Based on eight relatively small clinical observational studies, seven of which were retrospective in design, it has been meta‐analytically estimated that those who adhere to periodontal therapy would save approximately one tooth per 8 years compared to those who adhere to periodontal therapy erratically [3]. This estimate is considerably higher than that indicated by the findings of the present study. It was estimated that a person receiving periodontal therapy for five calendar years in a row would save an average of 0.08 (95% CI: 0.04; 0.13) teeth more than a similar person who received no periodontal therapy during those 5 years. This effect is quite small, considering that the data indicated an average of approximately 0.62 teeth (0.12 per year) lost in the five‐year period. Thus, in contrast to what has been expected in the literature [7], the rate of tooth loss seems to be relatively modest not only in regularly treated but also in non‐treated or erratically treated individuals. Notably, the estimated effect is based on a rather extreme contrast in the periodontal treatment pattern (no treatment in any of 5 years versus treatment in all 5 years), and thus reducing periodontal treatment frequency, for instance, to every second year, would likely not have a considerable effect on tooth loss compared to annual treatment frequency [17]. Even though the decade before age 40 is associated with the highest periodontitis incidence [39], it should be borne in mind that the cohort studied here was relatively young at the outset, 40 years. It might be speculated that the level of severity of periodontitis in this study sample has yet to reach a level where tooth extraction becomes a prominent treatment option. Certainly, it has been observed that the risk of tooth loss during periodontal maintenance is greater among the 60+ year‐olds [40]. In addition to differences in study design and definition of treatment adherence, a key difference between the present study and those included in the meta‐analysis [3] is that the present analysis adjusted for time‐invariant and time‐varying confounding, whereas the mentioned meta‐analytical estimates were calculated based on unadjusted data. However, it is an untenable assumption that those who adhere to periodontal care are similar to those who do not in all other periodontitis‐related aspects. While it is well‐known that those who receive periodontal therapy differ from those who do not in terms of their baseline characteristics [2, 41, 42, 43], it is less well recognised that the decision to opt for treatment at a certain time point is also influenced by past periodontal treatment experience and tooth loss, leading to time‐varying confounding with treatment‐confounder feedback over time. In such situations, methods like g‐estimation are required to properly estimate the treatment effect, and more traditional approaches are likely leading to biased treatment effect estimates because the effects of previous treatments or outcomes on current treatment or future outcomes are not adequately adjusted for [14, 15, 16, 26, 27, 28, 29].

In summary, this register‐based analysis showed that receiving periodontal therapy has a slightly reducing effect on tooth extractions 1–3 years ahead among 40‐year‐olds with incident periodontitis. While acknowledging the constraints of the study, these findings offer meaningful evidence on the real‐world effectiveness of periodontal therapy against tooth loss, which seems to be considerably smaller than indicated by earlier clinical studies [2, 3, 7, 8, 9, 10, 11]. Future research should focus on identifying subpopulations that would benefit most from regular periodontal care, helping to better allocate the scarce public resources available for oral health care, even in the world's wealthiest countries [44]. Moreover, research should explore the potential of utilising routinely collected healthcare and patient‐reported outcome measure data to estimate the impact of periodontal therapy on other patient‐important outcomes, such as orofacial function, pain, appearance, and psychosocial well‐being [45].

## Author Contributions

**Eero Raittio:** conceptualization; formal analysis; methodology; investigation; visualisation; writing – original draft. **Vibeke Baelum:** conceptualization; data curation; investigation; visualisation; project administration; supervision; writing – review and editing.

## Ethics Statement

Register‐based studies do not require individual informed consent. The study was approved by the Danish Data Protection Agency (2015‐57‐0002) and Aarhus University (2016–051–000001‐914).

## Consent

The authors have nothing to report.

## Conflicts of Interest

The authors declare no conflicts of interest.

## Supporting information


**Table S1:** Percentages of participants receiving individual prevention, supragingival treatment, subgingival treatment or surgical periodontal treatment between 2002 and 2020.
**Table S2:** Odds ratios (ORs) for receiving periodontal therapy within a calendar year from a logistic regression model, that is the propensity score model usen in g‐estimation.
**Figure S1:** The effect of receiving periodontal treatment within a calendar year on the number of extracted teeth (A, B) or probability of at least one tooth extraction (C) one to 5 years later.

## Data Availability

The data that support the findings of this study are available from Denmark Statistics. Restrictions apply to the availability of these data, which were used under license for this study.
