# Supplementary material for: Assessing the Impact of Periodontal Therapy on Tooth Loss: A Register‐Based Longitudinal Study in Denmark
Source: Community Dent Oral Epidemiol. 2025 Sep 26;53(6):724–36. doi: 10.1111/cdoe.70026 (PMC12627266; doi:10.1111/cdoe.70026)
Supplement: Supplementary file 1 — Table S1: Percentages of participants receiving individual prevention, supragingival treatment, subgingival treatment or surgical periodontal treatment between 2002 and 2020. Table S2: Odds ratios (ORs) for receiving periodontal therapy within a calendar year from a logistic regression model, that is the propensity score model usen in g‐estimation. Figure S1: The effect of receiving periodontal treatment within a calendar year on the number of extracted teeth (A, B) or probability of at least one tooth extraction (C) one to 5 years later. [file CDOE-53-724-s001.docx]

**SUPPLEMENTARY MATERIAL**

**Supplementary information about variables used in the analyses**

**Gender**

Men; Women. Information from: The Danish Civil Registration System (Pedersen 2011). Variable name in the register: KOEN.

**Origin**

Categorized into two groups: 1) Immigrants or descendants, and 2) persons of Danish origin. (Pedersen 2011). Variable name in the register: IE_TYPE

**Municipality of residence**

99 municipalities. Information from: The Danish Civil Registration System (Pedersen 2011). Variable name in the register: KOM.

**Highest completed education**

Categorized into eight groups:

• Primary school

• High school

• Vocational

• Short-cycle higher education or Qualifying exam

• Medium-cycle higher education

• Bachelor

• Long cycle higher education or researcher education

• Unknown

Information from the Danish Educational Register (Jensen et al. 2011). Categorized based on the variable in the register AUDD. Qualifying exam and Researcher education categories were combined with closest neighboring category due to their small size.

**Income percentile**

People were divided into 100 groups (percentiles) for each year from 1990 to 2021 based on their total annual personal income. Personal income in total is equal to the sum of business income, transfer income, property income (excluding calculated rental value of own home) and other non-classifiable income that can be attributed directly to the individual. The amount is before tax deduction, labor market contribution and special pension contribution, and interest expenses are not deducted. Information from the Danish Income Statistics Register (Baadsgaard et al. 2011). Variable name in the register: PERINDKIALT_13.

**Periodontal treatment (exposure)**

Any person selected as outlined above was defined to be exposed in the calendar year if the person had received (yes/no) periodontal care if they had received the following services:

| **Service** | **Codes** | **Details** |
| --- | --- | --- |
| Supragingival treatment | 1120, 1130, 2120, 1301, 1302, 1300 | Supragingival periodontal treatment (scale and polish) |
| Subgingival treatment of periodontal pockets less than 5 mm | 1420, 1425 | Subgingival periodontal treatment (instrumentation) and control |
| Subgingival treatment of periodontal pockets 5 mm or more | 1430, 1452, 1453, 1431 | Subgingival periodontal treatment (instrumentation) and control |
| Periodontal surgery | 1440, 1454 | Periodontal surgery and control |
| Individual prevention | 2920, 2930 | Individualized oral hygiene advice, fluoride application, smoking cessation advice, or dietary advice |

**Periodontal treatment history**

Periodontal treatment history was captured using a variable with the following levels: a) no periodontal treatment, b) individualized prevention or supragingival treatment (1120, 1130, 2120, 1301, 1302, 1300, 2920, 2930), c) subgingival periodontal treatment of periodontal pockets less than 5 mm (1420, 1425), d) subgingival or surgical periodontal treatment of deeper 5 mm or more periodontal pockets (1430, 1452, 1453, 1431, 1440, 1454). If a person received treatments from multiple categories, the highest level of complexity was assigned.

**Tooth extractions (outcome)**

The total number of treatment codes representing simple and surgical tooth extractions (1701, 1702, 1703, 1704, 1705,1801) were summed in each calendar year.

**Number of dental restorations per calendar year**

From the National Health Insurance Service Register (Sahl Andersen et al. 2011), we used the dental treatment codes that have been used over time since 1990 in the National Health Insurance scheme covering the subsidized dental care for all adult permanent residents in Denmark. A total number of treatment codes representing amalgam or composite restorations (1501, 1502, 1503, 1504, 1505, 1506, 1507, 1509, 1551, 1552, 1553, 1554, 1555, 1556, 1557, 1558, 1559) were summed in each calendar year.

**Incident diabetes mellitus type 1 or 2**

Data on incident diabetes between 1995 and 2021 comes from the Registry for Selected Chronic Diseases and Severe Mental Disorders (Danish Health Data Authority 2024). It combines information from the Danish National Prescription Registry (Pottegard et al. 2017), including all prescriptions in Denmark, and the Danish National Patient Registry (Schmidt et al. 2015) which contains information on all visits, procedures, and admissions to all Danish somatic hospitals, emergency departments, and hospital-associated outpatient clinics.

Individuals were classified as having incident diabetes mellitus (type 1 or 2) in a calendar year when one of the following criteria were met.

Diabetes type 1:

- Individuals registered with at least two purchases of insulin or insulin analogs (A10A, except combination medicines including GLP1-analogues and insulins, A10AE54 or A10AE56) in the Danish National Prescription Registry.
- Individuals registered with a relevant primary or secondary diagnosis (E10, diabetes type I, or its sub-codes under ICD-10) in the Danish National Patient Registry.

Diabetes type 2:

- Individuals registered with at least two purchases of medication aimed at lowering blood glucose (A10B, except A10BJ, A10BK01, A10BK03) or combination medicines including GLP1-analogues and insulins (A10AE54 or A10AE56) in the Danish National Prescription Registry.
- Individuals registered with a relevant primary or secondary diagnosis (E11, diabetes type 2, or its sub-codes under ICD-10) in the Danish National Patient Registry.

Excluded were:

- Women who have been exclusively treated with metformin (ATC code A10BA02) and there were signs that they could have polycystic ovary syndrome (prescription for G03GB02, G03HB or diagnosis code E282).
- Women who have a code for gestational diabetes (ICD-10 code O24.4) and who have only registered purchase of antidiabetics (A10) within 280 days before first contact or 280 days after last contact with gestational diabetes according to the Danish National Patient Registry.

**Number of teeth, number of filled teeth, and number of decayed teeth**

Since 2000, private dental practitioners have had to report the number of teeth, number of filled teeth, and number of decayed teeth for their patients turning 25, 40 or 65 years during the ongoing calendar year to the National Board of Health (Vilstrup et al. 2010) where these data have become integrated with the National Health Insurance Service Register. We extracted data on the number of teeth, number of filled teeth, and number of decayed teeth.

Data was cleaned as follows. If the recorded number of teeth was 0 (unlikely in 40-year-old Danes in 2001) or exceeded 32, data was considered as missing. Similarly, if recorded number of filled or number of filled decayed teeth exceeded 32, data was considered as missing. For all three variables values in the range from 29 to 32 were replaced with 28.

**Missing data**

Due to the way the sample was selected, the practically universal coverage of the registers, and the entitlement to subsidized dental care in Denmark, the extent of missing data in exposure, outcome, and covariates was rare in the sample. Probably because of lower coverage of the Educational Register, e.g., among immigrants (Jensen et al. 2011), 22 participants (1.8%) were assigned unknown/missing highest educational level. Otherwise, no missing data existed.

**Supplementary information on the statistical analysis**

**Lag effects from previous exposure, outcome and confounders**

The rationale behind the decision to allow a 1-to-5-year lag, rather than using all historical data, was to maintain computational efficiency without changing the primary estimate considerably. Another reason for allowing lagging effects only for outcome, exposure and restorative treatment history was that they had considerable time-varying aspect, and they were also key determinants of both outcome and exposure. Lagging effects for region, diabetes status, or highest educational attainment were excluded due to their rarity of change within the population, making their inclusion statistically redundant and analytically unsound. Similarly, lagging income effects were omitted, as the most recent income status was deemed the most important.

| **Time-varying confounder** | **Lag structure** | **Reason** |
| --- | --- | --- |
| Region | No lag | Insufficient variability over time |
| Highest completed education | No lag | Insufficient variability over time |
| Income percentile, splines | No lag | The most recent income is likely the most important |
| Diabetes, type 1 or type 2 | No lag | Insufficient variability over time |
| Number of restorations | 1-to-5-year lag | Caries is key reasons for tooth loss, and affects also periodontal treatment patterns |
| Periodontal treatment history (exposure history) | 1-to-5-year lag | Periodontal treatment history is key determinant of future periodontal treatment receival and tooth extractions |
| Number of extractions (outcome history) | 1-to-5-year lag | Previous extractions likely affect future periodontal treatment and extractions |

Principally, using the notation in the DAG (Figure 3), allowing a 1-to-5-year lag effect means that e.g., when investigating the effect of periodontal therapy in the sixth year, E6, on the number of extractions in the seventh year, O7, both the outcome model and the propensity score models include C, W6, V0-V5, E0-E5 and O0-O5. The same logic applies for all propensity score and outcome models in all other years. It is also worth noting that our approach allowed account of 1-to-5-year lag effects for E, O and V already from the beginning of the follow-up (2002, T=1) and onwards.

Table S1 represents the propensity score model used and shows also the variables included in it. Outcome models for each outcome timepoint (2003-2021) were identical, however they did not include the year variable.

**Performing G-estimation with** **gesttools package**

First, data was formatted according to guidance given for using gesttools package (Tompsett et al. 2022). Analyses were performed with gestMultiple function and bootsrapped with gestboost functions using following specifications. Outcome, propensity score, and censoring models included variables listed in Table S1. Outcome models also naturally included the exposure, receiving periodontal therapy in a calendar year. Time-varying effect (“type=3”) on tooth extractions five years ahead (“cutoff=5”) were estimated.

By default, the gesttools package works with binary and continuous outcomes (Tompsett et al. 2022). However, because count and binary outcomes can be estimated with the same procedure using gamma regression for outcome models (Dukes et al. 2018), we made minor modifications to the gesttools functions so that they can be used for count outcomes. R-scripts for these modified functions are available from GitHub and supplemented with validation analyses (<https://github.com/raittioe/gestCount>).

**Sensitivity analyses**

In the sensitivity analyses assuming the alternative directed acyclic graph (Figure 4), when estimating the effect from E6 to O7, the adjustment sets in propensity score and outcome models would change from C, W6, V0-V5, E0-E5 and O0-O5 to C, W6, V1-V6, E1-E5 and O1-O6, and similarly for all other combinations of exposure and outcome times.

**References**

Baadsgaard M, Quitzau J. 2011. Danish registers on personal income and transfer payments. Scand J Public Health. 39(7_suppl):103-105.

Danish Health Data Authority. 2024. Registry for selected chronic diseases and severe mental disorders [in danish].

Dukes O, Vansteelandt S. 2018. A note on g-estimation of causal risk ratios. Am J Epidemiol. 187(5):1079-1084.

Jensen VM, Rasmussen AW. 2011. Danish education registers. Scand J Public Health. 39(7_suppl):91-94.

Pedersen CB. 2011. The danish civil registration system. Scand J Public Health. 39(7_suppl):22-25.

Pottegard A, Schmidt SAJ, Wallach-Kildemoes H, Sorensen HT, Hallas J, Schmidt M. 2017. Data resource profile: The danish national prescription registry. 46(3):798-798f.

Sahl Andersen J, De Fine Olivarius N, Krasnik A. 2011. The danish national health service register. Scand J Public Health. 39(7_suppl):34-37.

Schmidt M, Schmidt SA, Sandegaard JL, Ehrenstein V, Pedersen L, Sorensen HT. 2015. The danish national patient registry: A review of content, data quality, and research potential. 7:449-490.

Tompsett D, Vansteelandt S, Dukes O, De Stavola BL. 2022. Gesttools: General purpose g-estimation in r. 8:1 - 28.

Vilstrup L, Christensen LB, Borge H, Kristensen SF. 2010. Oral health for users of private dental practice from 2000 to 2008 [in danish]. 114(9):704-712.

**Supplementary tables and figures**

**Table S1**. Percentages of participants receiving individual prevention, supragingival treatment, subgingival treatment or surgical periodontal treatment between 2002 and 2020.

| Year | Individual prevention | Supragingival treatment | Subgingival treatment, periodontal pockets less than 5 mm | Subgingival treatment, periodontal pockets 5 mm or more | Surgical periodontal treatment |
| --- | --- | --- | --- | --- | --- |
| 2002 | 10.5 | 57.6 | 27.5 | 17.5 | 0.7 |
| 2003 | 10.0 | 54.3 | 22.1 | 14.2 | 0.4 |
| 2004 | 9.6 | 53.2 | 22.4 | 14.8 | 0.6 |
| 2005 | 9.7 | 52.7 | 22.8 | 15.3 | 0.8 |
| 2006 | 10.5 | 48.2 | 22.5 | 14.4 | 0.3 |
| 2007 | 11.3 | 51.0 | 24.2 | 16.0 | 0.4 |
| 2008 | 11.0 | 48.7 | 25.1 | 16.9 | 0.3 |
| 2009 | 10.7 | 48.2 | 25.1 | 16.9 | 0.2 |
| 2010 | 9.5 | 45.7 | 24.6 | 18.3 | 0.2 |
| 2011 | 9.4 | 46.4 | 26.0 | 19.1 | 0.5 |
| 2012 | 9.7 | 45.7 | 25.5 | 18.0 | 0.3 |
| 2013 | 11.0 | 33.4 | 27.9 | 19.4 | 0.3 |
| 2014 | 15.0 | 31.6 | 29.6 | 20.1 | 0.4 |
| 2015 | 28.2 | 38.9 | 27.4 | 22.7 | 0.4 |
| 2016 | 34.3 | 38.9 | 26.9 | 24.6 | 0.3 |
| 2017 | 32.6 | 39.5 | 25.9 | 24.1 | 0.2 |
| 2018 | 31.2 | 39.1 | 25.3 | 25.3 | 0.1 |
| 2019 | 29.6 | 38.9 | 26.8 | 26.8 | 0.3 |
| 2020 | 24.0 | 35.9 | 25.3 | 25.4 | 0.1 |

**Table S2**. Odds ratios (ORs) for receiving periodontal therapy within a calendar year from a logistic regression model, that is the propensity score model usen in g-estimation.

OR 2.5% 97.5% z val.

Intercept 0.05 0.03 0.09 -10.20

Gender (ref. Women)

Men 0.81 0.75 0.87 -5.40

Danish origin (ref. No)

Yes 1.31 1.15 1.51 3.93

Region (ref. North Jutland)

Central Jutland 0.84 0.73 0.98 -2.26

Southern Denmark 0.87 0.75 1.01 -1.87

Zealand 0.85 0.74 0.98 -2.20

Greater Copenhagen 0.88 0.75 1.02 -1.66

Number of teeth at baseline 1.01 1.00 1.02 1.32

Number of filled teeth at baseline, splines

1^st^ spline 0.87 0.73 1.03 -1.60

2^nd^ spline 0.89 0.62 1.27 -0.65

3^rd^ spline 1.22 0.92 1.61 1.37

Number of decayed teeth at baseline 0.98 0.97 0.99 -3.64

Highest completed education (ref. Unknown)

Primary school 1.45 1.04 2.01 2.19

High school 1.60 1.11 2.30 2.53

Vocational 1.69 1.21 2.34 3.10

Short-cycle higher education or Qualifying exam 1.86 1.30 2.66 3.39

Medium-cycle higher education 1.81 1.28 2.55 3.39

Bachelor 1.25 0.80 1.95 1.00

Long cycle higher education or researcher education 1.92 1.32 2.79 3.42

Income percentile, splines

1^st^ spline 1.58 1.36 1.84 5.86

2^nd^ spline 1.68 1.14 2.49 2.59

3^rd^ spline 1.41 1.22 1.63 4.64

Diabetes, type 1 or type 2 (ref. No)

Yes 0.99 0.83 1.19 -0.09

Periodontal therapy in previous 5 years

1-year lag (ref. no periodontal treatment)

Individualized prevention or supragingival treatment 4.60 4.18 5.06 31.08

Treatment of periodontal pockets <5mm 6.24 5.41 7.18 25.35

Treatment of periodontal pockets ≥5mm 8.11 7.13 9.23 31.83

2-year lag (ref. no periodontal treatment)

Individualized prevention or supragingival treatment 2.62 2.37 2.91 18.38

Treatment of periodontal pockets <5mm 2.39 2.06 2.78 11.35

Treatment of periodontal pockets ≥5mm 2.29 1.99 2.63 11.66

3-year lag (ref. no periodontal treatment)

Individualized prevention or supragingival treatment 1.89 1.70 2.10 11.87

Treatment of periodontal pockets <5mm 1.82 1.56 2.13 7.54

Treatment of periodontal pockets ≥5mm 2.03 1.75 2.35 9.46

4-year lag (ref. no periodontal treatment)

Individualized prevention or supragingival treatment 1.59 1.43 1.77 8.62

Treatment of periodontal pockets <5mm 1.40 1.19 1.64 4.11

Treatment of periodontal pockets ≥5mm 1.48 1.27 1.73 5.09

5-year lag (ref. no periodontal treatment)

Individualized prevention or supragingival treatment 1.74 1.57 1.92 10.79

Treatment of periodontal pockets <5mm 1.92 1.65 2.25 8.24

Treatment of periodontal pockets ≥5mm 1.82 1.57 2.11 7.93

Number of restorations in previous years

1-year lag 1.03 1.01 1.06 2.51

2-year lag 1.03 1.00 1.06 2.29

3-year lag 1.02 0.99 1.04 1.13

4-year lag 0.98 0.95 1.00 -1.87

5-year lag 0.97 0.95 0.99 -2.30

Number of extractions in previous years

1-year lag 1.00 0.95 1.05 -0.11

2-year lag 0.95 0.90 1.01 -1.73

3-year lag 0.92 0.87 0.98 -2.57

4-year lag 0.98 0.92 1.04 -0.79

5-year lag 0.94 0.88 1.00 -1.87

Year (ref. 2002)

2003 1.01 0.78 1.31 0.07

2004 1.08 0.83 1.41 0.60

2005 1.16 0.89 1.52 1.13

2006 0.76 0.59 0.98 -2.11

2007 1.25 0.98 1.59 1.80

2008 1.30 1.02 1.65 2.10

2009 1.20 0.94 1.53 1.48

2010 1.07 0.84 1.36 0.53

2011 1.29 1.01 1.65 2.07

2012 1.10 0.86 1.41 0.79

2013 0.64 0.51 0.82 -3.62

2014 0.81 0.64 1.03 -1.75

2015 1.18 0.93 1.50 1.35

2016 1.14 0.89 1.45 1.06

2017 1.17 0.92 1.50 1.28

2018 1.13 0.89 1.45 0.99

2019 1.09 0.85 1.39 0.67

2020 0.77 0.61 0.99 -2.07


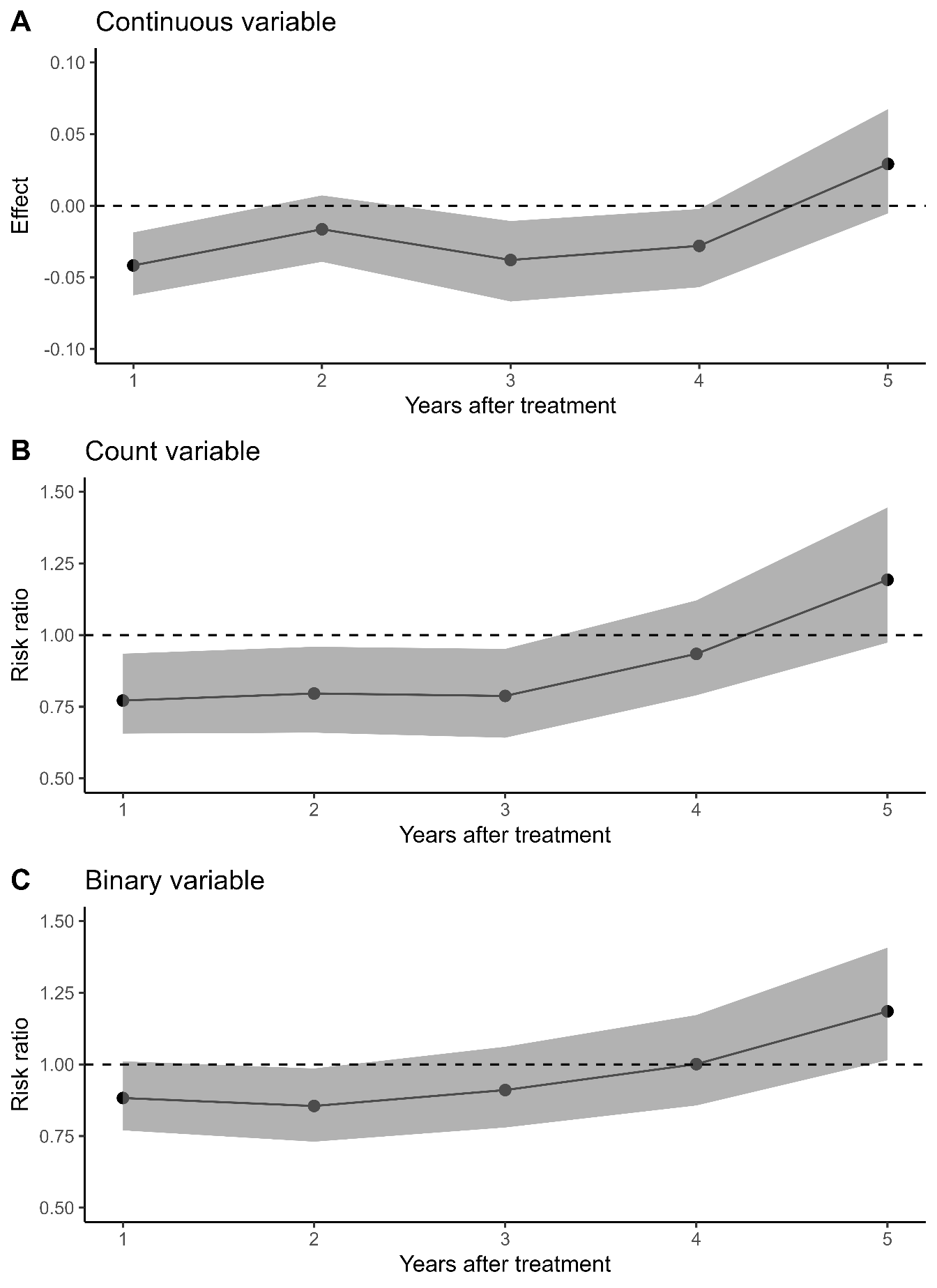


**Figure S1.** The effect of receiving periodontal treatment within a calendar year on the number of extracted teeth (A, B) or probability of at least one tooth extraction (C) one to five years later. The right panel shows the effect of receiving periodontal treatment within a calendar year in those with severe or non-severe condition at the baseline. **Adjustment set selected based on the alternative DAG (Figure 4).**
